# Influence of Frailty on Clinical and Radiological Outcomes in Patients Undergoing Transforaminal Lumbar Interbody Fusion—Analysis of a Controlled Cohort of 408 Patients

**DOI:** 10.3390/jcm14061814

**Published:** 2025-03-07

**Authors:** Yesim Yildiz, Stefan Motov, Felix Stengel, Lorenzo Bertulli, Gregor Fischer, Linda Bättig, Francis Kissling, Laurin Feuerstein, Daniele Gianoli, Thomas Schöfl, Michael G. Fehlings, Benjamin Martens, Martin N. Stienen, Nader Hejrati

**Affiliations:** 1 Department of Neurosurgery, Cantonal Hospital of St. Gallen, 9000 St. Gallen, Switzerland; 2 Spine Center of Eastern Switzerland, Cantonal Hospital of St. Gallen, 9000 St. Gallen, Switzerland; 3 Department of Orthopedic Surgery, Cantonal Hospital of St. Gallen, 9000 St. Gallen, Switzerland; 4 Division of Neurosurgery and Spine Program, Department of Surgery, University of Toronto, Toronto, ON M5T 1P5, Canada

**Keywords:** transforaminal lumbar interbody fusion, frailty, Canadian Frailty Index, cage subsidence, complications, outcome

## Abstract

**Background/Objectives**: The concept of frailty has been recognized as an important issue which can influence postoperative outcomes. We aimed to investigate the influence of frailty on clinical and radiological outcomes in patients undergoing transforaminal lumbar interbody fusion (TLIF) for degenerative spine disease. **Methods**: A single-center, retrospective cohort study was conducted involving 408 patients in whom 506 expandable interbody devices were implanted. The patients were grouped into vulnerable/frail versus well/fit according to the Canadian Frailty Index. **Results**: The frail patients were older and had a larger number of fused segments (3.0 vs. 2.4 segments, *p* = 0.009). In the univariate analysis, the frail patients were more likely to experience a postoperative adverse event (AE) until discharge (OR 1.89, 95% CI 1.22–2.92; *p* = 0.004), three (OR 1.57, 1.07–2.3; *p* = 0.021), and 12 months postoperatively (OR 3.77, 1.96–7.24; *p* < 0.001). Following the multivariable logistic regression analysis, frailty remained an independent risk factor for postoperative AEs at 12 months (OR 3.44, 95% CI 1.69–6.99; *p* = 0.001). **Conclusions**: Frailty negatively influenced the rate of AEs until 12 months, while the odds of having a favorable outcome at any time remained unaffected in patients undergoing posterior spinal fusion with TLIF. Future efforts are needed to evaluate whether preoperative medical optimization or prehabilitation may positively impact patient outcomes.

## 1. Introduction

Based on the aging population and overall increasing life expectancy, clinicians will be faced with more age-related disorders of the spine. Over the years, spinal fusion surgeries have increased at a higher frequency than other notable in-patient procedures, and the average patient age has also increased for these procedures [1]. With these developments, concerns are being raised that elderly patients with comorbidities could be at increased risk for complications and worse outcomes after spinal fusion procedures.

Considering this, the concept of frailty has become increasingly recognized as an important issue which can influence clinical outcomes [2]. Frailty differentiates between chronological and physiological aging, as it describes a decrease in functional reserve capacities, which may be caused by the interaction of progressive age-related decline in physiological systems on the one hand and an increase in chronic diseases on the other [3]. It is generally defined as a state of vulnerability to stressors, which may be endogenous or exogenous, thereby increasing the risk of negative health-related outcomes [4]. Due to different definitions of frailty status, the prevalence of who can be considered a “frail patient” varies widely between studies. And yet, a correlation with increasing age has been described. While a higher prevalence of degenerative spine diseases is found within the elderly patient population, complex spine surgeries may offer relief, but they may also carry a higher risk in frail patients, thereby negatively influencing perioperative morbidity [5].

The literature on the relevance of frailty in patients undergoing transforaminal lumbar interbody fusion (TLIF) surgery is currently limited. TLIF is a frequently performed surgical procedure that utilizes a posterolateral transforaminal approach to the disk, allowing for either an open or a minimally invasive (MIS) decompression and 360° fusion of a spinal motion segment. Higher levels of frailty have been shown to be predictive of readmission, reoperations, any complications, and adjacent segment disease in older patients undergoing single-level TLIF [6]. Open TLIF in frail patients is also associated with an increased revision rate and the probability to be discharged to a location other than home [7]. Cage subsidence has been shown to be a potential complication of decompression and fusion surgeries, with age and low bone mineral density (BMD) being significantly associated with worse results, factors which pertain to the frail patient population [8]. Moreover, cage subsidence may lead to a loss of lumbar lordosis, potentially affecting sagittal alignment parameters negatively. However, to the best of our knowledge, the impact of frailty on cage subsidence—and its subsequent effects on clinical and radiological outcomes following TLIF surgery—has not yet been investigated. With the growing number of frail patients requiring TLIF surgery, this topic remains increasingly relevant.

## 2. Materials and Methods

### 2.1. Study Type & Patient Identification

This was a retrospective, single-center observational controlled cohort study including patients from the Spine Center of Eastern Switzerland, Cantonal Hospital St. Gallen, Switzerland. Patients who received a TLIF procedure for degenerative disease (disk herniation, spinal stenosis with suspected instability, degenerative spondylolisthesis, isthmic spondylolisthesis, and others) were included. All patients received either single- or multi-level fusion procedures with an Altera^®^ Expandable Integrated TLIF Spacer (Globus Medical Inc., Audubon, PA 19403, USA) between October 2018 and September 2023. Grafts that were used included local bone graft, iliac crest graft, and/or allogen graft. Patients were identified by electronic review of our hospital’s purchasing department’s records, and the operating program was additionally cross-checked.

### 2.2. Data Collection

A retrospective review of the electronic medical records with extraction of relevant information was performed by 11 surgeons. A comprehensive codebook outlining the definition and classification of all variables was developed prior to data collection to ensure consistency and accuracy. Data collection was standardized through clear instructions on score calculation and measurements, which were uniformly distributed among all raters. An independent spinal surgeon (MNS) conducted regular audits to ensure data accuracy and quality, adhering to the standards. The demographic data included age, sex, body mass index (BMI; in kg/m^2^) and smoking status. We also included the American Society of Anesthesiologists (ASA) grading scale of surgical risk, the Charlson Comorbidity Index (CCI), and the Canadian Frailty Index (CFI) (see Supplementary Table S1), which were prospectively collected by our anesthesiologists [9,10].

At our hospital, anesthesiologists are trained to document ASA, CCI, and CFI scores for all patients undergoing general anesthesia across various medical and surgical disciplines. These scores are subsequently reviewed by board-certified anesthesiologists to ensure accuracy. Surgical parameters, such as the number of fused segments and cement augmentation of pedicle screws, were recorded. Intraoperatively, the decision to perform cement augmentation of screws was made by a board-certified spinal orthopedic surgeon or neurosurgeon, based on the intraoperative evaluation of the screw purchase. Adverse events (AEs) were assessed intraoperatively, at time of discharge, and three and 12 months after surgery and were classified in severity according to the Therapy-Disability-Neurology (TDN) scoring system [11]. Cage subsidence (CS) was assessed on intraoperative X-ray imaging, standing lateral and anterior-posterior X-ray imaging at discharge, and at three- and 12-month follow-up and was defined as a breach into the endplates. Hounsfield units (HU) of the involved segments were recorded and measured as the average HU of the above and below vertebra on sagittal and axial views (i.e., average of four values), (Supplementary Figure S1). Further radiological parameters included the degree of intersomatic fusion according to the Brantigan, Steffee, and Fraser (BSF) classification, the degree of posterolateral fusion according to Lenke et al. (rated on postoperative computed tomography [CT] scans, whenever available), as well as further spinopelvic parameters at three and 12 months after surgery [12,13]. Clinical outcomes were graded according to the MacNab criteria into four categories (excellent, good, fair, or poor), as best estimated from the follow-up letter and the electronic patient chart [14].

### 2.3. Frailty Score & Stratification

The CFI is a seven-point grading scale which was developed in 2005 and ranks patients from very fit to severely frail [10]. It is a simple tool that allows for the assessment of a patient’s degree of frailty and level of fitness. Based on the CFI grading, we separated the patients into two groups. The first group included patients who were “managing well”, “well”, or were “very fit”, and will hereafter be referred to as the “non-frail” group. The second group included patients who were “vulnerable”, “mildly frail”, “moderately frail”, or “severely frail” and will hereafter be referred to as the “frail” group. The CCI predicts one- and 10-year-survival based on age and a set of 19 common comorbidities, thereby allowing for an objective assessment of patients [9].

### 2.4. Statistical Analysis

For the statistical analyses, Stata SE (StataCorp LLC, College Station, TX, USA) v18.0 for Mac was used. We mostly employed descriptive statistics, reporting results as mean (standard deviation; SD) or count (percent). Probability values of <0.05 were considered statistically significant. A uni- and multivariable logistic regression model was used for analysis of the surgical AEs and outcomes at discharge and after three and 12 months and calculating odds ratios (ORs) and 95% confidence intervals (CIs), adjusting for the following potentially confounding variables: age, smoking status, disease type, CCI, and ASA grade after ruling out collinearity. Sensitivity analyses were performed with slightly different stratifications of the CFI score (e.g., excluding “vulnerable” patients in the frail group).

### 2.5. Ethical Considerations

The study was approved by the institutional review board of Eastern Switzerland (BASEC ID 2023-01343). All research was performed in accordance with relevant guidelines and regulations. Retrospective collection, analysis, and publication of anonymized patient data was allowed with an institutional waiver for informed consent.

## 3. Results

A total of n = 408 patients were identified, who underwent TLIF surgery on 506 levels. 56.1% (n = 229) were female. The non-frail group consisted of n = 276 (67.6%) and the frail group of n = 132 patients (32.4%). The mean age of patients in the frail group was significantly higher (69.2 years vs. 63.6 years, *p* < 0.001). There were no significant differences in the gender distribution. Not surprisingly, frail patients were more likely to have higher ASA grades (*p* < 0.001) and higher CCI scores (*p* < 0.001). Complete baseline demographic information is depicted in Table 1.

Regarding surgery-specific variables, the overall extent of fusion (mono-/bisegmental vs. multi-level fusion; *p* = 0.002) and number of segments fused during the index surgery (2.4 [SD 2.3] vs. 3.0 [SD 2.5], *p* = 0.009) was higher in frail patients. Cement augmentation of pedicle screws was more commonly performed in frail patients (33.3% vs. 16.3%, *p* < 0.001). No significant differences were observed in the other surgery-specific variables, including the segment at which a TLIF was performed, the length of the procedure, estimated blood loss, and intraoperative AEs (Table 2).

Frail patients had a longer length of stay (LOS; 12.3 vs. 10.2 days, *p* = 0.016), and a higher rate of AEs was detected in frail patients until discharge (40.1% vs. 26.4%, *p* = 0.005), as well as at three (25.0% vs. 10.9%, *p* = 0.001) and 12 months of follow-up (20.4% vs. 6.5%, *p* < 0.001; Table 3). While no significant difference in the type of AEs (i.e., medical or surgical) was observed at all time points, AEs in frail patients were found to be more often severe (as classified by the TDN score) at three (*p* = 0.004) and 12 months follow-up (*p* = 0.001). There were no significant differences regarding the degree of intersomatic and posterolateral fusion and presence of clinically relevant pseudarthrosis at any time between both groups (Table 3). However, CS was diagnosed significantly more often in the frail group at time of discharge (21.5% vs. 15.2%, *p* = 0.037) while there was a trend toward higher rates of CS at three (33.1% vs. 24.5%, *p* = 0.09) and 12 months postoperatively (33.1% vs. 23.9%, *p* = 0.075). Clinical outcomes were inferior in the frail patients both at the three- (*p* < 0.001) and the 12-month follow-up (*p* = 0.044).

In the univariate logistic regression (Table 4), frail patients were more likely to experience a postoperative AE until discharge (OR 1.89, 95% CI 1.22–2.92) and at three (OR 1.57, 95% CI 1.07–2.3) and 12 months of follow-up (OR 3.77, 95% CI 1.96–7.24) while the odds of experiencing a favorable outcome at any time postoperatively remained unaffected. The multivariable logistic regression, adjusted for confounders and baseline differences including age, CCI, ASA grades, and numbers of segments fused, showed that frailty was an independent risk factor for postoperative AEs at 12 months (OR 3.44, 95% CI 1.69–6.99), while keeping a tendency for inferior clinical outcomes (OR 0.70, 95% CI 0.44–1.1, Table 4). The sensitivity analyses showed no significant differences in the overall results and robustness of the linear regression models.

In terms of spinopelvic parameters (Table 5), frail patients started with more dysbalanced spines preoperatively, represented by lower total lumbar lordosis (LL; 45.9° vs. 50.6°, *p* = 0.006), a higher C7 sagittal vertical axis (C7 SVA; 7.0 vs. 4.8 cm, *p* = 0.004), and a tendency for a more pronounced mismatch between ideal and actual mismatch (11.5° vs. 8.5°, *p* = 0.055). Before discharge, total LL in frail patients was corrected to the extent that no statistically significant differences were observed between the frail and the non-frail patients (LL; 50.6° vs. 50.4°, *p* = 0.898). In the postoperative course, total LL significantly decreased in the frail patients at three months (LL; 50.8° vs. 54.4°, *p* = 0.011) and 12 months postoperatively (LL; 51.2° vs. 54.5°, *p* = 0.032). This resulted in a higher PI–LL mismatch (8.6° vs. 3.6°, *p* = 0.006) and in a higher mismatch between ideal and actual LL (8.9° vs. 3.6°, *p* = 0.004).

### Illustrative Case

**Figure 1:**
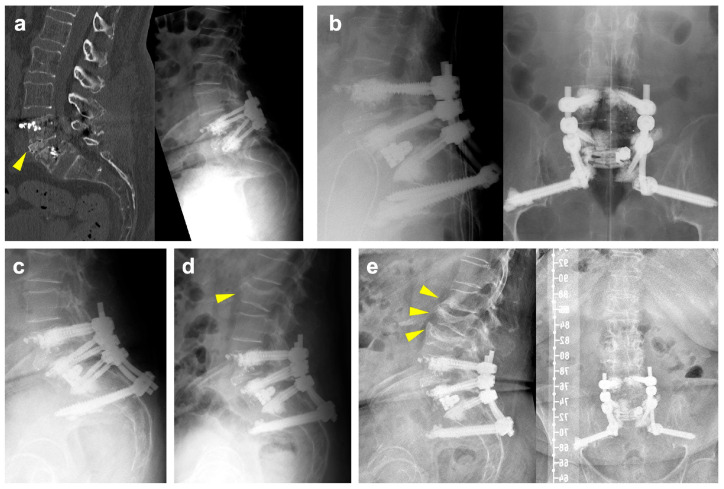
(**a**): Preoperative CT scan (left) and standing radiographs (right) demonstrating a burst/split fracture of the L5 vertebra and cage subsidence into the lower endplate of L4 (yellow arrowhead). (**b**): Intraoperative lateral (left) and ap (right) radiographs following cement-augmented revision surgery involving a TLIF with an expandable interbody spacer at L5/S1 and extension of the construct to the pelvis. (**c**): Postoperative lateral radiographs demonstrating slight progression of cage subsidence into to L5 vertebral body. (**d**): Lateral radiograph following readmission showing a new fracture of the superior endplate of L2 (yellow arrowhead). (**e**): Lateral (left) and ap (right) radiographs at 6 months of follow-up illustrating new fractures of the lower endplate of L1, an incomplete burst fracture of L3 and progression of the L2 fracture (yellow arrowheads).

The patient is a 71-year-old female with a previous history of degenerative spondylolisthesis at L4/5 with central spinal canal stenosis and associated symptoms of spinal claudication. She therefore underwent TLIF surgery at L4/5 and bilateral decompression at L5/S1 one year prior to the current admission. At the one-year follow-up, she presented with an exacerbation of lumbar pain radiating to the right lower extremity, which was attributed to an adjacent segment degeneration at L5/S1 and a burst fracture of the L5 vertebra (Figure 1a). Clinically manifest osteoporosis with insufficiency fractures were previously known. Medical treatment with vitamin D and denosumab was previously initiated. Due to unbearable pain, revision surgery was proposed, and extension of the construct distally into the pelvis with TLIF at L5/S1 was planned. Unfortunately, the procedure had to be postponed multiple times due to episodes of cardiac decompensation. As assessed by our anesthesiologists, the preoperative CCI grade was 1 and the ASA grade was 3. Ultimately and following preoperative cardiac optimization, the intervention was performed without intraoperative complications (Figure 1b). Postoperative imaging revealed cranial cage subsidence (Figure 1c). Despite this, the patient reported a significant reduction in pain compared to preoperatively. During hospitalization, she developed anemia, which was managed with transfusion of red blood cells. Moreover, she tested positive for influenza. On postop day nine, the patient was discharged to rehabilitation.

A few days after discharge, she was readmitted to cardiology due to subacute myocardial infarction and pulmonary edema. Following an episode of coughing accompanied by sudden onset of back pain, imaging revealed a new fracture of the superior endplate of L2, which did not require surgical intervention (Figure 1d). At her six-month follow-up, she reported persistent right-sided lumbar pain radiating to the buttock, although improved compared to her preoperative status. Imaging at that time showed new osteoporotic fractures at L1 and L3 (Figure 1e). Subsequently, the patient refused further follow-up at our clinic due to the long distance from her residence. She currently remains in follow-up with her general practitioner.

## 4. Discussion

Taking into consideration the increasing age of the global population, clinicians will be faced with more spinal disorders specific to the elderly and the aging of the spine [15]. To the best of our knowledge, there is a paucity of literature on the influence of frailty on clinical and radiological outcomes in patients undergoing TLIF. Moreover, this is the largest study where expandable interbody spacers were applied—a novel but increasingly used technology. In this study, we were able to demonstrate that frailty has a significant and clinically meaningful negative impact on the likelihood of developing postoperative AEs, while clinical patient outcomes remain influenced to a lesser extent, losing the statistical significance after adjusting for multiple confounding variables. Frailty is certainly an important issue for spine surgeons to be aware of, and the problem we face today is likely to become even more important in the future. Similar to our results in TLIF patients, prior studies have shown that frail and pre-frail patients undergoing posterior lumbar interbody fusion are at higher risk of major complications, readmissions, prolonged hospital stays, and postoperative infections [16,17]. Additionally, in anterior lumbar interbody fusion procedures, frailty has been identified as an independent predictor of pulmonary complications, urinary tract infections, pneumonia, and unplanned intubations [18,19]. These findings highlight the consistent impact of frailty across different spinal fusion techniques, reinforcing the need for careful preoperative risk assessment and optimization. Topics like meticulous patient selection, including, for example, baseline expectations and psychological capacity to handle unsatisfactory results, as well as patient counseling, including setting appropriate expectations, reviewing the potential risks of surgery, postoperative instructions to limit complications, and long-term follow-up, become increasingly relevant [20]. Moreover, our current results highlight the need to address factors that may be influenced actively before admitting a patient for surgery, e.g., preoperative medical optimization or prehabilitation [21].

Our study characterized frail patients to be significantly older than non-frail patients. More importantly, both CCI and ASA grades were significantly higher in the frail patients, which is likely to further contribute to patients’ perioperative vulnerability. Recognition of a patient’s individual vulnerability and initiation of appropriate measures is therefore of utmost importance:

(1) Prehabilitation programs, consisting of preoperative measures, such as general education, exercise, pain education, health behavior counseling, and mindfulness, have been shown to be feasible, reduce medical costs and improve postoperative pain, reduce disability, and improve satisfaction with surgical outcomes [22].

(2) Multidisciplinary perioperative management approaches, such as the Enhanced Recovery After Surgery (ERAS) approach, include different measures, such as patient education, risk assessment screening, usage of long-lasting local anesthetics, avoidance of urinary catheterization, opioid-limited or opioid-free analgesia, early postoperative mobilization, and prompt nutrition, and have been shown to reduce complications, readmissions, and LOS and improve functional recovery as well as patient-reported outcomes in spine surgery [23].

(3) Several studies have previously highlighted the importance of postoperative rehabilitation for patients undergoing spinal surgery. However, there is a paucity of studies that specifically focus on frail patients. In elderly patients undergoing lumbar fusion surgery due to degenerative intervertebral disk disease, systemic lower limb rehabilitation led to faster recovery, increased patient satisfaction rate and lesser lower limb deep venous thrombosis [24]. Also, the ERAS protocol showed that elderly frail patients undergoing multi-level lumbar fusion surgery had significant improvements regarding the recovery of physiological functions and reduction of hospital length of stay [23]. These findings highlight the importance of future research in rehabilitation protocols for frail patients.

(4) Finally, patient selection and counseling are imperative, as it has previously been shown that a preoperative discrepancy between outcome expectation and actuality led to lower patient satisfaction [25].

Future studies should also explore the impact of baseline mobility, pain levels, and patient-reported quality of life on outcomes following spinal surgery [12].

In our frail cohort, patients were not only significantly older but also included a higher proportion of females. Increased age and female sex are known risk factors for the development of osteoporosis. In the presence of conditions characterized by low BMD, such as osteoporosis, cement augmentation of pedicle screws may be indicated in spinal fusion procedures to reduce the risk of hardware failure [26]. As a result, our findings showed that frail patients were significantly more likely to undergo cement augmentation of pedicle screws than non-frail patients.

Our results further highlight higher rates of CS in frail patients. Age has been identified as a risk factor for the occurrence of CS in patients undergoing open and MIS-TLIF surgery [27,28,29,30]. This may partially be due to age-related comorbidities and changes in bone metabolism, as well as the use of medication affecting BMD, ultimately resulting in osteoporosis [31]. Low BMD and osteoporosis, along with cage shape, size, and position, axial compression load of the cage–endplate interface, the amount of cartilaginous endplate removal during surgery, or endplate injury have been identified as risk factors for the occurrence of CS [32,33]. Interestingly, the occurrence of CS did not significantly affect the degree of intersomatic fusion or the rate of clinical pseudarthrosis between the frail and the non-frail patients undergoing TLIF surgery. In line with our findings, previous studies have shown that cage subsidence does not necessarily need to be associated with a lower fusion rate or worse clinical outcomes [34,35]. We hypothesize that our surgical approach with rigorous removal of the inferior facet joints and cartilage removal provides an additional basis for secondary posterior fusion, as evidenced by the non-significant differences in posterolateral fusion rates between the groups. Ultimately, while preoperative assessment of BMD in patients undergoing spinal fusion surgery is crucial, this is particularly true for patients who are classified as frail. In this retrospective study, we did not collect BMD measurement in all patients, but information on the mean HUs of the adjacent vertebrae was collected and showed no significant differences between the frail and the non-frail patients. Irrespective of the BMD status, however, expandable interbody spacers may be associated with a higher risk of CS [36]. This has been ascribed to the high forces exerted on the endplates during the expansion process, especially if the segmental release is not thoroughly performed before. Expansion of the interbody spacer should be halted as soon as a good press fit is achieved in order to avoid a breach into the endplates. Ultimately, as seen within our cohort, CS may be one potential reason for the loss of LL on follow-up.

Our study revealed a significantly longer LOS in frail patients, which may partly be explained by the larger extent of the surgery. Frail patients had significantly larger constructs, which consequently harbor a higher probability of risks, complications, and worse outcomes. In line with our findings, previous studies have shown that frail patients undergoing TLIF had a longer LOS and a lesser probability to be discharged home while being more often discharged to locations other than home, such as rehabilitation centers or nursing homes [7,16,37,38,39]. Another explanation for the prolonged LOS seen in frail patients may be the significantly higher occurrence and severity of postoperative AEs. Consistent with our results, previous studies have shown that frailty was significantly associated with the occurrence of postoperative AEs in patients undergoing TLIF [6,16,39,40]. Our univariable analysis indicates that patients were more likely to experience AEs at any time after surgery. However, this finding may reflect significant baseline differences in comorbidities, as measured by the CCI, as well as the extent of the fusion procedure between the frail and the non-frail patients. After adjustment, frail patients were found to be more likely to experience an AE at 12 months postoperatively. And yet, while frail patients in our study were 3.4 times as likely as the non-frail patients to experience a postoperative AE at 12 months, logistic regression analysis did not demonstrate a significant impact of frailty on the odds of having a favorable 12-month outcome (OR 0.71, 95% CI 0.49–1.04, *p* = 0.078). Previous studies were able to detect a negative impact of frailty on the clinical outcomes with statistical significance. A retrospective study analyzing N = 488 patients undergoing one- or two-level TLIF surgery showed that severely frail patients had fewer improvements in back pain, with age and CCI also being predictors for inferior outcomes in terms of back and leg pain [39]. It is important to acknowledge, however, that the latter study focused on severely frail patients only, whereas our frail patient group consisted of a more heterogenous population spanning moderate and severe degrees of frailty.

A possible explanation for the comparable favorable outcome in the frail and the non-frail patients after three and 12 months may be found in the sagittal balance measurements, as it is widely accepted that spinopelvic alignment is important for and correlates with postoperative outcomes [41]. A positive sagittal imbalance leads to an increased workload on accessory muscles to stay erect during gait and therefore leads to early fatigue and pain especially in the back, buttocks, and thighs [42]. The C7 SVA has previously been shown to be related to clinical outcomes after spinal fusion surgeries and to be an indicator of health-related quality of life [43]. Accordingly, in our study, SVA was significantly different between frail and non-frail patients preoperatively, as there were more patients with ASD in the frail cohort. However, there were no significant differences found after three and 12 months, which may explain the similar postoperative outcomes in the multivariate analysis. In line with our findings, a prospective, multi-center, and multi-continental study previously showed that patients over 60 years of age who underwent multi-level spinal fusion had significant improvements in the Oswestry Disability Index after successful procedures, demonstrating that the elderly population may also benefit from ASD surgery, considering age-adjusted alignment goals [44].

Given our findings, counseling frail patients undergoing TLIF surgery should emphasize their higher likelihood of postoperative complications, including cage subsidence. However, it is important to note that these complications, which often resolve over time, do not necessarily impact long-term outcomes compared to non-frail patients. Therefore, TLIF surgery remains a viable option that can be offered to frail patients if appropriately indicated, with consideration of preoperative optimization strategies to enhance their functional capacity.

### Strengths and Limitations

The strengths of this study include the large cohort of consecutively treated patients by a variety of surgeons, as well as a reasonably low missing data burden. The reason for missing data at the three and 12 months follow-up involves the inclusion of patients whose surgery was not long enough ago at the time of the data collection. Three-month follow-up data were available in n = 387 patients (94.9%) and 12-month follow-up data in n = 313 patients (76.7%). Our study revealed clear results with a strikingly high effect size for a topic that is relevant already today, but will be even more important in the future considering the aging population.

The retrospective nature of this study along with its limitations may be considered a weakness. A fourth of patients received another type of interbody fusion in addition to the TLIF during the same procedure, which makes it difficult to establish a direct effect of the intra- or postoperative AEs or the clinical outcome to the TLIF procedure. On the other hand, being inclusive and considering the typical patient cohort of a public, academic spine center, makes our results more generalizable regarding the influence of frailty in spinal (fusion) procedures. Another limitation is the stratification of patients into two groups (frail and non-frail patients), which was decided based on clinical reasoning and was necessary to employ statistical methods like multivariable logistic regression. Sensitivity analyses with slightly modified stratifications of study groups did not reveal much different results. Analyzing each CFI grade separately would have required a much larger sample to keep sufficiently high statistical power. As the results of this study parallel our clinical observations and make sense, we think that having included even “vulnerable” or “mildly frail” patients in the frail cohort is justified.

Moreover, certain surgery-related factors, such as the surgeon’s experience and the chosen surgical approach, were not evaluated in this study and may have a potential influence on outcomes. The use of a single implant type limits the generalizability of our findings to other types of interbody spacers. However, it provided a controlled study environment where the influence of only one specific interbody spacer was examined, thereby reducing the risk for potential confounding. Finally, even though not significantly different between the groups, frail patients had longer operative times and greater blood loss on average, which may have introduced confounding. However, since the operative time and blood loss have shown collinearity with the numbers of segments fused, our multivariable analysis was adjusted for the numbers of segments fused.

## 5. Conclusions

Frailty has a significant and clinically meaningful negative impact on the likelihood of developing an AE within the first year after TLIF. Our results, however, demonstrate that this seems to influence long-term clinical outcomes to a lesser extent. Comprehensive, multidisciplinary patient care, meticulous counseling, preoperative optimization of medical comorbidities such as osteoporosis, as well as prehabilitation and ERAS protocols, are likely to positively influence AE rates and outcomes in frail patients, which should be examined in further studies.

## Figures and Tables

**Table 1:** Patient demographics of N = 408 patients undergoing thoracolumbar fusion surgery including the use of an Altera^®^ expandable interbody spacer on 506 levels further stratified by CFI categories of well to fit (i.e., managing well, well, or very fit) and vulnerable to frail (i.e., vulnerable, mildly frail, moderately frail, or severely frail).

Variables	Categories		*p*-Value
	Well to Fit (n = 276)	Vulnerable to Frail (n = 132)	
Age, in years	63.6 (SD 13.2)	69.2 (SD 10.6)	**<0.001**
Sex Female Male	149 (54%) 127 (46%)	80 (60.6%) 52 (39.4%)	0.207
BMI categories * Underweight Healthy Overweight Obese	3 (1.1%) 92 (33.3%) 95 (34.4%) 86 (31.2%)	0 (0%) 31 (23.4%) 47 (35.6%) 54 (40.97%)	0.079
Smoking status Nonsmoker Smoker Former smoker	160 (57.9%) 78 (28.3%) 38 (13.8%)	76 (57.6%) 47 (35.6%) 9 (6.8%)	0.070
ASA grade I II III IV	11 (4.0%) 180 (65.2%) 82 (29.7%) 3 (1.1%)	0 (0%) 42 (31.8%) 84 (63.6%) 6 (4.6%)	**<0.001**
CCI Very low Mild Moderate Severe	116 (42.0%) 104 (37.7%) 39 (14.1%) 17 (6.2%)	33 (25.0%) 54 (40.9%) 24 (18.2%) 21 (15.9%)	**0.001**
CFI Very fit Managing well Vulnerable Mildly frail Moderately frail Severely frail	24 (8.7) 109 (39.5%) 143 (51.8%) na na na na	na na na 103 (78.0%) 19 (14.4%) 7 (5.3%) 3 (2.3%)	na
Average Hounsfield Unit of the upper and lower vertebra †	166.2 (SD 73.3)	162 (SD 76.8)	0.593
Total	N = 408 (100%) patients and 506 (100%) segments		

Demographic data are presented as count (percent) or mean (standard deviation; SD). * BMI categories are according to the WHO classification (<18.5 kg/m^2^: underweight; 18.5–24.9 kg/m^2^: healthy; 25–30 kg/m^2^: overweight; >30 kg/m^2^: obese). † Hounsfield units were calculated as an average of the upper and lower instrumented vertebra on axial and sagittal computed tomography images (Supplementary Figure S1). ASA = American Society of Anesthesiology; BMI = body mass index; CCI = Charlson Comorbidity Index; CFI = Canadian Frailty Index; WHO = World Health Organization. Statistically significant results (*p* < 0.05) were marked in bold font.

**Table 2:** Surgery-specific information of N = 408 patients undergoing thoracolumbar fusion surgery including the use of an Altera^®^ expandable interbody spacer on 506 levels further stratified by CFI categories of well to fit (i.e., managing well, well, or very fit) and vulnerable to frail (i.e., vulnerable, mildly frail, moderately frail, or severely frail).

Variables	Categories		*p*-Value
	Well to Fit (n = 276)	Vulnerable to Frail (n = 132)	
TLIF segment ** Th12/L1 L1/2 L2/3 L3/4 L4/5 L5/S1	1 (0.3%) 6 (1.7%) 11 (3.2%) 41 (12.0%) 152 (44.3%) 132 (38.5%)	2 (1.2%) 6 (3.7%) 9 (5.5%) 24 (14.7%) 60 (36.8%) 62 (38.1%)	0.219
Extent of fusion * Mono-/bi- segmental 3–7 segments 8 or more segments	202 (73.2%) 57 (20.6%) 17 (6.2%)	74 (56.1%) 47 (35.6%) 11 (8.3%)	**0.002**
Number of fused segments	2.4 (SD 2.3)	3.0 (SD 2.5)	**0.009**
Type of interbody spacer 8° lordotic 15° lordotic	169 (49.3%) 174 (50.7%)	77 (47.2%) 86 (52.8%)	0.669
Other types of interbody fusion employed * None XLIF/LLIF ALIF PLIF Other	210 (76.1%) 23 (8.3%) 23 (8.3%) 17 (6.2%) 3 (1.1%)	102 (77.3%) 12 (9.1%) 5 (3.8%) 10 (7.6%) 3 (2.2%)	0.421
Cement augmentation of pedicle screws * No Yes	231 (83.7%) 45 (16.3%)	88 (66.7%) 44 (33.3%)	**<0.001**
Type of laminectomy ** Partial Complete	254 (74.1%) 89 (25.9%)	123 (75.5%) 40 (24.5%)	0.734
Length of surgery, in minutes	305 (SD 132)	319 (SD 129)	0.316
EBL, in milliliters	765 (SD 859)	815 (SD 585)	0.541
Intraoperative AEs † No Yes	238 (86.2%) 38 (13.8%)	112 (84.9%) 20 (15.1%)	0.708
Intraoperative cage subsidence ** No Yes Missing data/unclear	315 (91.8%) 24 (7.0%) 4 (1.2%)	149 (91.4%) 14 (8.6%) 0 (0%)	0.320
Total	N = 408 (100%) patients and 506 (100%) segments		

Surgery-specific data are presented as count (percent) or mean (standard deviation; SD) on a patient-level (*) or on a segment level (**). † Type of intraoperative AEs included: n = 36 incidental durotomy (8.9%), n = 8 excessive bleeding (2.0%), n = 6 hardware-related (1.5%), n = 5 osseous injury (1.2%) and n = 1 pleural injury (0.3%). AE = adverse event; ALIF = anterior lumbar interbody fusion; EBL = estimated blood loss; LLIF = lateral lumbar interbody fusion; PLIF = posterior lumbar interbody fusion; TLIF = transforaminal lumbar interbody fusion; XLIF: extreme lateral interbody fusion. Statistically significant results (*p* < 0.05) were marked in bold font.

**Table 3:** Information on surgical adverse events (AEs) and outcome at discharge, 90 days, and 12 months in N = 408 patients undergoing thoracolumbar fusion surgery including the use of an Altera^®^ expandable interbody spacer in N = 506 segments further stratified by CFI categories of well to fit (i.e., managing well, well, or very fit) and vulnerable to frail (i.e., vulnerable, mildly frail, moderately frail, or severely frail).

Variable	Discharge			90 Days			12 Months		
	Well to Fit (n = 276)	Vulnerable to Frail (n = 132)	*p*-Value	Well to Fit (n = 261; 94.6%)	Vulnerable to Frail (n = 126; 95.5%)	*p*-Value	Well to Fit (n = 210; 76.1%)	Vulnerable to Frail (n = 103; 78.0%)	*p*-Value
Length of stay/mean follow-up	10.2 (SD 8)	12.3 (SD 8.8)	**0.016**	88.1 (SD 35)	92.5 (SD 50)	0.307	370 (SD 105.4)	365 (SD 114.1)	0.626
AE † No Yes Missing	203 (73.6%) 73 (26.4%) 0 (0.0%)	79 (59.9%) 53 (40.1%) 0 (0.0%)	**0.005**	229 (83.0%) 30 (10.9%) 17 (6.1%)	91 (68.9%) 33 (25.0%) 8 (6.1%)	**0.001**	191 (69.2%) 18 (6.5%) 67 (24.3%)	76 (57.6%) 27 (20.4%) 29 (22.0%)	**<0.001**
AE type Medical Surgical	49 (69.0%) 22 (31.0%)	39 (75.0%) 13 (25.0%)	0.467	2 (6.7%) 28 (93.3%)	2 (6.1%) 31 (93.9%)	0.922	0 (0%) 18 (100%)	1 (3.7%) 26 (96.3%)	0.409
TDN grade of AE 1 2 3 4 5 Missing data	4 (1.5%) 33 (12.0%) 24 (8.7%) 9 (3.3%) 2 (0.7%) 0 (0.0%)	5 (3.8%) 24 (18.3%) 14 (10.7%) 8 (6.1%) 1 (0.8%) 0 (0.0%)	0.103	2 (0.7%) 1 (0.4%) 25 (9.1%) 2 (0.7%) 0 (0.0%) 17 (6.2%)	6 (4.5%) 3 (2.3%) 22 (16.7%) 2 (1.5%) 0 (0.0%) 8 (6.1%)	**0.004**	1 (0.4%) 2 (0.7%) 14 (5.1%) 1 (0.4%) 0 (0.0%) 67 (24.3%)	0 (0.0%) 4 (3.0%) 22 (16.7%) 0 (0.0%) 1 (0.8%) 29 (22.0%)	**0.001**
Clinical outcome Excellent Good Fair Poor Missing data	n/a	n/a	n/a	99 (34%) 105 (36.1%) 49 (16.9%) 19 (6.5%) 19 (6.5%)	20 (15.1%) 62 (47.0%) 22 (16.7%) 20 (15.1%) 8 (6.1%)	**<0.001**	86 (31.2%) 71 (25.7%) 38 (13.8%) 11 (4.0%) 70 (25.3%)	32 (24.2%) 30 (22.7%) 27 (20.5%) 13 (9.9%) 30 (22.7%)	**0.044**
Posterolateral fusion Definitely not solid Probably not solid Possibly solid Definitely solid Missing/ unclear	n/a	n/a	n/a	10 (3.6%) 120 (43.5%) 44 (15.9%) 13 (4.7%) 89 (32.3%)	6 (4.6%) 65 (49.2%) 26 (19.7%) 5 (3.8%) 30 (22.7%)	0.339	10 (3.6%) 41 (14.9%) 63 (22.8%) 56 (20.3%) 106 (38.4%)	5 (3.8%) 24 (18.2%) 44 (33.3%) 21 (15.9%) 38 (28.8%)	0.106
Intersomatic fusion Fusion Interme diate type Pseudar throsis Missing/ unclear	n/a	n/a	n/a	8 (2.9%) 162 (58.7%) 18 (6.5%) 88 (31.9%)	4 (3.0%) 83 (62.9%) 14 (10.6%) 31 (23.5%)	0.228	90 (32.6%) 66 (23.9%) 21 (7.6%) 99 (35.9%)	36 (27.3%) 45 (34.1%) 14 (10.6%) 37 (28.0%)	0.078
Clinical pseudarthrosis No Yes Missing/unclear	n/a	n/a	n/a	221 (80.1%) 15 (5.4%) 40 (15.5%)	105 (79.6%) 9 (6.8%) 18 (13.6%)	0.844	187 (67.8%) 18 (6.5%) 71 (25.7%)	86 (65.2%) 12 (9.1%) 34 (25.7%)	0.713
Cage subsidence * No Yes Missing data/unclear	283 (82.5%) 52 (15.2%) 8 (2.3%)	128 (78.5%) 35 (21.5%) 0 (0%)	**0.037**	227 (66.2%) 84 (24.5%) 32 (9.3%)	92 (56.4%) 54 (33.1%) 17 (10.4%)	0.090	179 (52.2%) 82 (23.9%) 82 (23.9%)	71 (43.6%) 54 (33.1%) 38 (23.3%)	0.075

Data are presented as count (percent) or mean (standard deviation). n/a = not applicable; TDN = Therapy-Disability-Neurology. “Posterolateral fusion was assessed according to the Lenke classification as (0) definitively not solid (graft resorption or obvious pseudarthrosis, (1) probably not solid (small, thin fusion masses bilaterally), (2) possibly solid (unilateral large fusion mass) and (3) definitively solid (bilateral, big trabecular fusion masses).” [13]. “Intersomatic fusion was assessed according to the Brantigan, Steffee und Fraser-Classification for intersomatic fusion in the former disk space.” [12]. “Clinical pseudarthrosis was defined when patients suffered from typical axial or radicular pain weeks or months after the surgery after exclusion of other reasons for pain”. † Type of AEs at discharge: n = 38 anemia (9.4%), n = 19 wound healing disorder/infection/hematoma (4.7%), n = 16 pulmonary embolism (3.9%), n = 11 new neurological deficit/neuropathic pain (2.7%), n = 10 other AEs (2.5%), n = 9 congestive heart failure (2.2%), n = 6 urinary tract infection (1.5%), n = 5 delirium (1.2%), n = 4 renal failure (1.0%), n = 3 fracture/hardware-related issues (0.7%), and n = 3 pneumonia (0.7%). Type of AEs at 90 days: n = 23 fracture/hardware-related issues (5.6%), n = 18 wound healing disorder/infection/hematoma (4.4%), n = 18 adjacent segment disease/proximal junctional kyphosis or failure (4.4%), each n = 1 renal failure (0.3%), pulmonary embolism (0.3%), congestive heart failure (0.3%), and pneumonia (0.3%). Type of AEs at 12 months: n = 24 adjacent segment disease/proximal junctional kyphosis or failure (5.9%), n = 9 pseudarthrosis (2.2%), n = 6 fracture/hardware-related issues (1.5%), n = 5 wound healing disorder/infection/hematoma (1.2%), and n = 1 pulmonary embolism (0.3%). * Rates are reported based on the segment level where cage subsidence was observed. Statistically significant results (*p* < 0.05) were marked in bold font.

**Table 4:** Logistic regression model of surgical adverse events (AEs) and outcomes at discharge, 90 days, and 12 months in patients categorized as “well to fit” (managing well, well, or very fit) versus “vulnerable to frail” (vulnerable, mildly frail, moderately frail, or severely frail) according to the Canadian Frailty Index. Analysis was computed for N = 408 patients undergoing thoracolumbar fusion surgery, including the use of an Altera^®^ expandable interbody spacer in N = 506 segments. The multivariable analysis was adjusted for the following variables: frailty, age, CCI and ASA, and the number of segments fused.

Outcome of Interest	Univariable Analysis			Adjusted Analysis		
	OR	95% CI	*p*-Value	OR	95% CI	*p*-Value
Intraoperative AEs	1.12	(0.62–2.071)	0.710	0.88	(0.47–1.66)	0.693
Postoperative AEs until discharge	1.89	(1.22–2.92)	**0.004**	1.04	(0.62– 1.74)	0.893
Postoperative AEs at 90 days	1.57	(1.07–2.3)	**0.021**	1.6	(0.94–2.72)	0.084
Postoperative AEs at 12 months	3.77	(1.96–7.24)	**<0.001**	3.44	(1.69–6.99)	**0.001**
Favorable outcome at 90 days	0.77	(0.53–1.12)	0.167	0.75	(0.46–1.2)	0.230
Favorable outcome at 12 months	0.71	(0.49–1.04)	0.078	0.7	(0.44–1.1)	0.123

Data are presented as odds ratio (OR) with 95% confidence interval (CI). Notes: Age was dichotomized into <65 and ≥65 years. Favorable outcome was defined as MacNab classification of excellent/good versus fair/poor. ASA: American Society of Anesthesiologists; CCI: Charlson Comorbidity Index. Statistically significant results (*p* < 0.05) were marked in bold font.

**Table 5:** Information on spinopelvic parameters and intervertebral distance in N = 408 patients undergoing thoracolumbar fusion surgery including the use of an Altera^®^ expandable interbody spacer in 506 segments further stratified by CFI categories well to fit (i.e., managing well, well, or very fit) and vulnerable to frail (i.e., vulnerable, mildly frail, moderately frail, or severely frail).

Spinopelvic Parameters	Preoperative			Discharge			90 Days Postoperative			12 Months Postoperative		
	Well to Fit	Vulnerable to Frail	*p*-Value	Well to Fit	Vulnerable to Frail	*p*-Value	Well to Fit	Vulnerable to Frail	*p*-Value	Well to Fit	Vulnerable to Frail	*p*-Value
PI, in °	58.3 (13.3)	57.0 (12.9)	0.387	n/a	n/a	n/a	n/a	n/a	n/a	n/a	n/a	n/a
Total LL, in °	50.6 (15.8)	45.9 (15.5)	**0.006**	50.4 (13.2)	50.6 (10.8)	0.898	54.4 (13)	50.8 (11.3)	**0.011**	54.5 (12.9)	51.2 (12)	**0.032**
SS, in °	38.3 (10.8)	36.4 (10.6)	0.087	37.5 (9.6)	37.6 (10)	0.963	39.6 (10)	36.5 (10)	**0.005**	39.4 (9.9)	37.9 (9.7)	0.213
PT, in °	19.8 (9.7)	20.7 (10.1)	0.397	20.2 (11)	19.7 (11.1)	0.633	19.2 (11.4)	21.7 (13.4)	0.056	18.8 (12.2)	20.9 (11.2)	0.150
Segmental lordosis, in ° *	17.1 (9.7)	16.2 (10.3)	0.349	21.4 (9)	20.7 (9.4)	0.439	20.7 (8.7)	19.5 (9.2)	0.170	19.8 (9)	18.8 (8.8)	0.271
C7 SVA, in cm	4.8 (4.3)	7.0 (6.1)	0.004	4.4 (3.8)	5.7 (4.4)	0.204	5.5 (5)	5.3 (3.9)	0.888	5.8 (6.4)	5.5 (5)	0.857
Roussouly type			**0.049**			n/a			n/a			**n/a**
1	17 (6.1)	16 (12.1)										
2	35 (12.7)	8 (6.1)										
3	98 (35.5)	52 (39.4)										
4	123 (44.6)	56 (42.4)										
Missing/unclear	3 (1.1)	0 (0)										
Ideal LL, in °	58.7 (8.0)	57.5 (7.5)	**0.152**			n/a			n/a			n/a
Ideal–actual LL mismatch, in °	8.5 (15.0)	11.5 (15.2)	**0.055**	7.8 (13.1)	7.3 (12.1)	0.705	5.1 (14.7)	7.8 (14.2)	0.094	3.9 (13.9)	8.9 (16)	**0.004**
PI-LL mismatch, in °	7.9 (14.4)	11.1 (16.9)	**0.051**	7.4 (14)	6.8 (13.9)	0.720	5 (15.4)	7.4 (15.8)	0.156	3.6 (14.9)	8.6 (15.9)	**0.006**

Data are presented as count (percent) or mean (standard deviation), comparing measurements at follow-up with preoperative. LL = lumbar lordosis, C7 SVA = C7 sagittal vertical axis, PI = pelvic incidence, PT = pelvic tilt, SS = sacral slope, n/a = not applicable. Statistically significant results (*p* < 0.05) were marked in bold font. * Segmental lordosis was calculated per operated level.

## Data Availability

The datasets generated and analyzed during the current study are available from the corresponding author on reasonable request.

## Supplementary Materials

The following supporting information can be downloaded at: https://www.mdpi.com/article/10.3390/jcm14061814/s1, Figure S1: Exemplary demonstration of the calculation of the Houndsfield Unit (HU) for the level L4/L5; Table S1: Canadian Frailty Index.

## Abbreviations

The following abbreviations are used in this manuscript:

AE	Adverse events
ASA	American Society of Anesthesiologists
ASD	Adult spinal deformity
BMD	Bone mineral density
BMI	Body mass index
BSF	Brantigan, Steffee, and Fraser (classification)
CCI	Charlson Comorbidity Index
CFI	Canadian Frailty Index
CS	Cage subsidence
ERAS	Enhanced Recovery After Surgery
HU	Hounsfield Unit
LOS	Length of stay
MIS	Minimally invasive surgery
SVA	Sagittal vertical axis
TDN	Therapy-Disability-Neurology (scoring system)
TLIF	Transforaminal lumbar interbody fusion
