# Supplementary material for: Influence of Frailty on Clinical and Radiological Outcomes in Patients Undergoing Transforaminal Lumbar Interbody Fusion—Analysis of a Controlled Cohort of 408 Patients
Source: J Clin Med. 2025 Mar 7;14(6):1814. doi: 10.3390/jcm14061814 (PMC11942930; doi:10.3390/jcm14061814)
Supplement: Supplementary file 1 [file jcm-14-01814-s001.zip › jcm-3472202-supplementary.pdf]

SUPPLEMENTARY INFORMATION

Supplementary Figure S1

An exemplary demonstration of the calculation of Hounsfield Units (HU) at the L4/5 level. HU of the involved segments were recorded and measured as the average HU values from both the superior and inferior vertebrae, measured on sagittal and axial views (i.e. average of four values).

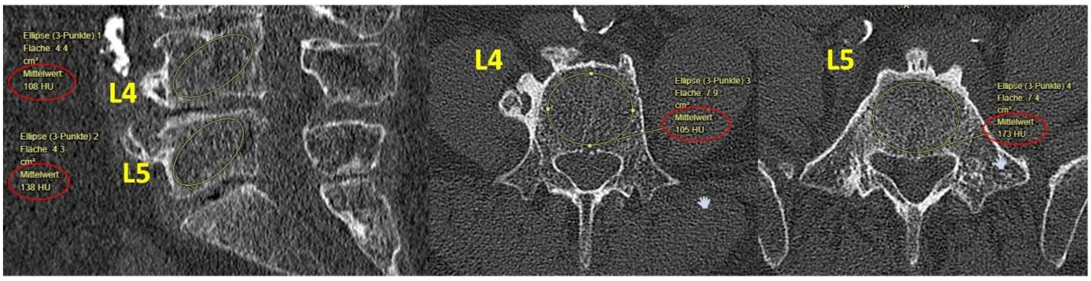

Supplementary Table S1: Canadian Frailty Index*

**Grade**		**Description**
1	Very fit	Robust, active, energetic, well-motivated and fit. These people commonly exercise regularly and are in the most fit group for their age.
2	Well	Without active disease, but less good than people than people in category 1.
3	Well, with treated comorbid disease	Disease symptoms are well controlled compared with those in category 4.
4	Apparently vulnerable	Although not frankly dependent, these people commonly complain of “being slowed up” or have disease symptoms.
5	Mildly frail	With limited dependence on others for instrumental activities of daily living.
6	Moderately frail	Help is needed with bot instrumental and non-instrumental activities of daily living.
7	Severely frail	Completely dependent on others for the acitivites of daily living or terminally ill.

* Rockwood K, Song X, MacKnight C, et al. A global clinical measure of fitness and frailty in elderly people. *CMAJ*. Aug 30 2005;173(5):489-95. doi:10.1503/cmaj.050051
